# A Role for Neuropilins in the Interaction between Schwann Cells and Meningeal Cells

**DOI:** 10.1371/journal.pone.0109401

**Published:** 2014-10-14

**Authors:** Kasper C. D. Roet, Kerstin T. S. Wirz, Elske H. P. Franssen, Joost Verhaagen

**Affiliations:** 1 Laboratory for Neuroregeneration, Netherlands Institute for Neuroscience, an Institute of The Royal Academy of Arts and Sciences, Amsterdam, The Netherlands; 2 Department of Molecular and Cellular Neurobiology, Center for Neurogenomics and Cognition Research, Vrije Universiteit Amsterdam, Amsterdam, The Netherlands; Centro de Investigación Príncipe Felipe – CIPF, Spain

## Abstract

In their natural habitat, the peripheral nerve, Schwann cells (SCs) form nicely aligned pathways (also known as the bands of Büngner) that guide regenerating axons to their targets. Schwann cells that are implanted in the lesioned spinal cord fail to align in pathways that could support axon growth but form cellular clusters that exhibit only limited intermingling with the astrocytes and meningeal cells (MCs) that are present in the neural scar. The formation of cell clusters can be studied in co-cultures of SCs and MCs. In these co-cultures SCs form cluster-like non-overlapping cell aggregates with well-defined boundaries. There are several indications that neuropilins (NRPs) play an important role in MC-induced SC aggregation. Both SCs and MCs express NRP1 and NRP2 and SCs express the NRP ligands Sema3B, C and E while MCs express Sema3A, C, E and F. We now demonstrate that in SC-MC co-cultures, siRNA mediated knockdown of NRP2 in SCs decreased the formation of SC clusters while these SCs maintained their capacity to align in bands of Büngner-like columnar arrays. Unexpectedly, knockdown of NRP1 expression resulted in a significant increase in SC aggregation. These results suggest that a reduction in NRP2 expression may enhance the capacity of implanted SCs to interact with MCs that invade a neural scar formed after a lesion of the spinal cord.

## Introduction

In 1980 and 1981, two studies reported significant regenerative growth of injured central nervous system fibers into peripheral nerve segments that were implanted in the lesioned rat spinal cord [1], [2]. It is now generally accepted that Schwann cells (SCs) are responsible for promoting axonal regeneration into peripheral nerve implants [3], [4]. The observations of Aguayo and Richardson and colleagues led to a new area of spinal cord injury (SCI) research which focused on understanding the pro-regenerative properties of SCs.

SCs can be readily obtained from peripheral nerves and can be expanded in culture. This allows autologous transplantation and makes them suitable cells for clinical transplantation strategies [5], [6]. Numerous studies have reported beneficial effects of SCs after implantation in the injured spinal cord, including increased axonal regeneration, remyelination of nerve fibers, decreased tissue loss and modest functional improvements [3], [5], [7]–[13].

After implantation in the lesioned spinal cord, SCs exhibit only limited migration and intermingling with host spinal cord cells [14]. Thus, implanted SCs remain largely concentrated at the site of deposition resulting in a relatively well-defined boundary between the transplanted SCs and non-permissive spinal cord scar tissue [14]–[16]. Knowledge of the molecular mechanisms responsible for the formation of this boundary could potentially be used to promote migration of SCs into the neural scar thereby rendering the scar tissue more permissive for axon regeneration [17].

Astrocytes and meningeal cells (MCs) are the two major cellular components of the scar after a SCI [10]. Currently, most studies have focused on the interaction of SCs with astrocytes after implantation or in cell culture [15], [18], [19] and several molecular mechanisms that govern this interaction, including N-cadherin mediated adhesion, Eph-ephrin signaling and aggrecan-integrin interactions [15], [20]–[22], have been described. We have shown that SCs form clusters when confronted or co-cultured with MCs [23]. The development of strategies that interfere with this cluster formation may result in improved SC integration in the inhospitable spinal cord scar tissue and thereby improve the formation of axon growth supporting SC bridges after implantation in the transected spinal cord.

There are several indications that class 3 semaphorins and their receptors neuropilin-1 and neuropilin-2 (NRP1–2) [24]–[26] mediate the migratory behavior of SCs in the injured spinal cord. Semaphorin3A (Sema3A) expression is increased in MCs in the spinal cord after damage [27]–[29]. Administration of a Sema3A inhibitor following a spinal cord lesion resulted in an increase of peripheral-type myelination of regenerated axons and a strong reduction of cavity formation [30]. These effects may result from increased endogenous SC infiltration from the dorsal roots into the lesion site. SCs do express the Sema3A receptor NRP1, and cultured SCs avoid areas where Sema3A is present, an effect that is relieved in the presence of a Sema3A inhibitor [30]. Furthermore, NRP2 function is important for SC alignment in vitro [31] and after peripheral nerve damage NRP1 and NRP2 expression are both increased in SCs [32], [33].

In this study we tested the hypothesis that the interaction between MCs and SCs is mediated by NRP1 and/or NRP2. We used an interaction assay to study MC-SC behavior in co-culture following selective knock-down of NRP1 or NRP2 in SCs. Unexpectedly, NRP1 and NRP2 knock-down had differential effects on MC-induced SC aggregation. Knockdown of NRP1 promoted SC cluster formation while knockdown of NRP2 resulted in a decreased formation of SC clusters and an increase in loosely aligned bundles of SCs.

## Materials and Methods

### Cell cultures

All animal experimental procedures were approved by the animal welfare committee of the Royal Netherlands Academy of Sciences. MC and SC cultures were prepared from inbred adult female Fischer 344 (180–220 gram, 8 to 10 weeks of age; Harlan, The Netherlands). The animals were deeply anesthetized with CO_2_ and decapitated. SCs were prepared from sciatic nerves as described previously [4], [23]. The sciatic nerves were dissected and the epineurial sheaths were removed in Leibovitz-15 medium (L15; Invitrogen). Subsequently, the nerves were cut in segments of 1–2 mm and cultured on plastic in Dulbecco’s modified Eagle’s medium (DMEM; Invitrogen) supplemented with 10% fetal calf serum (FCS; Invitrogen) and 1% penicillin/streptomycin (PS; Invitrogen) (D-10S). For 5 consecutive weeks, the sciatic nerve explants were replated every 7 days to allow fibroblasts to migrate out of the explants. After 6 weeks, no fibroblasts could be observed and the explants were incubated overnight at 37°C in 0.05% collagenase (Invitrogen) in D-10S. The next day, the explants were centrifuged at 290 g for 3 min, dissociated in D-10S supplemented with 2 µM forskolin (Sigma-Aldrich) and 20 µg/ml pituitary extract (PEX, Sigma-Aldrich) (D-10SFP) and plated in dishes coated for 2 h at 37°C with 20 µg/ml poly-L-lysine (PLL; Sigma-Aldrich). After 3 days of culturing, SCs were either used for experiments or were frozen in DMEM containing 30% FCS, PS, forskolin, PEX and 10% DMSO (Sigma Aldrich) and stored in liquid nitrogen. This established SC culture procedure results in a purity of >99% based on P75 labeling [23].

MCs were isolated from the meninges covering the olfactory bulbs. Meninges were incubated in HBSS, containing 0.125% trypsin, 0.5 mM EDTA and 0.25% collagenase for 45 min at 37°C in 5% CO_2_. Trypsinization was stopped by adding D-10S. After centrifugation for 3 min at 201 g, the pellet was resuspended in D-10S. The meninges were triturated through a 5 ml pipette, followed by a syringe with 19G and 25G needles. MCs were plated on PLL-coated 10 cm^2^ dishes. Previous characterization of this established MC culture procedure indicated that the MCs in culture are a pure cell population and express a very similar profile of marker proteins as described in vivo in the normal brain and the injured brain based on the presence of retinaldehyde dehydrogenase type 2 and the absence of glial fibrillary acidic protein labeling [28].

### Transfection

SCs were transfected with siGENOME SMART pools for NRP1 or NRP2 (Dharmacon) consisting of 4 siRNA duplexes per gene to a final concentration of 100 nM. Transfection was performed according to the manufacturer’s protocol, using DharmaFECT 3. In short, transfection mix was prepared by mixing siRNA and DharmaFECT 3, both diluted in serum-free medium. After incubation for 20 min at room temperature, pre-warmed (37°C) medium containing 0.5% FCS was added. Culture medium was removed from the cells and replaced by transfection mix. After 4 h, transfection mix was removed and replaced by culture medium. Individual siRNAs from the SMART pools were tested at a concentration of 100 nM each. When NRP1 and NRP2 SMART pools were combined, each pool had a final concentration of 50 nM.

### Interaction and confrontation assay

Interaction assays and confrontation assays were performed as previously described with small modifications [19], [23].

#### Interaction assay

On day 1 SCs were plated on PLL-coated 96-wells plates with a density of 8×10^3^ cells/well in D-10SFP. The following day the cells were transfected with siRNA as described above. Two days later, MCs were added to the SCs with a density of 8000 cells/well in D-10SFP, and cultures were fixed 5 days later.

#### Confrontation assay

SCs were plated in a drop of ca. 15 µl, containing 2×10^6^ cells/ml, on PLL-coated coverslips. MCs were plated in another drop, containing 1.2×10^6^ cells/ml, directly adjacent to the drop of SCs on the same coverslip. Both the SC and the MC drop were spread into thin adjacent lines with a 10 µl pipette tip. After 4 hours, unattached cells were removed by washing with medium and cells were maintained in DF-10SFP. After 5 days of culturing, cells were fixed and confrontation assays were analyzed as described below.

### Immunocytochemistry

Cultures were fixed with 4% PFA for 30 min. Fixed cells were rinsed and blocked for 30 min in PBS containing 0.1% Triton X-100 and 2% FCS (PBS-TS). Cells were incubated with S100 pAb (1∶600, Dako) in PBS-TS for 2 hr at room temperature (RT), washed with PBS, and incubated with a Cy2- or Cy3-labeled secondary antibody (1∶400; Jackson) in PBS-TS for 2 hr at RT. Nuclei were labeled with Hoechst 33258 (Invitrogen; 1∶1000). Coverslips were washed and mounted in Mowiol. Images were captured by fluorescence microscopy.

### Quantification of cell clusters

S100 positive SC cluster formation in the presence of MCs was quantified using the Morphology Bioapplication on the KineticScan High Content Screening (HCS) Reader (Cellomics) as previously described and visualized [23]. The complete surface area of each well was imaged in 9 non-overlapping fields in an automated fashion. Using the Cellomics Morphology Bioapplication, cell clusters in each field were automatically outlined and the area of each cluster was determined together with the total number of clusters in the 9 fields. Total cluster area per well was calculated by adding up all measured cluster areas per well. Parameters of the bioapplication were set before starting each screen by visually inspecting the results after running the algorithm and parameters were adjusted if necessary to trace clusters accurately. For each condition, at least 2 wells were measured per experiment. Well averages were normalized to the siGlo condition per experiment. All experiments were repeated at least 3 times and the presented results are the normalized average of all wells of all experiments for each condition. Conditions were compared using the Student’s t-test.

### mRNA quantification

To determine the knockdown efficiency of NRP1 and NRP2, SCs were plated and transfected in 12-well plates (2.1×10^5^ cells/well). The time course and the concentration of the transfection reagents were the same as in the aggregation assays. Cells were lyzed with Trizol (Invitrogen) and 20% chloroform was added. After centrifugation at 1.2×10^3^ g and 4°C for 15 min, the aqueous phase was removed, mixed with an equal volume of 70% ethanol and loaded on Rneasy Mini kit columns (Qiagen). RNA was isolated according to the manufacturer's protocol. cDNA was synthesized with a QuantiTect Reverse Transcription Kit (Qiagen). Real-time RT-PCR was used to quantify relative mRNA levels using SYBR GREEN PCR Master Mix (Applied Biosystems). Beta-actin, RPS24 and EF1alpha were used as reference genes. Expression levels were compared using the Student’s t-test.

## Results

### Schwann cells and meningeal cells express both neuropilins as well as a distinct set of class 3 semaphorins

**Figure 1:**
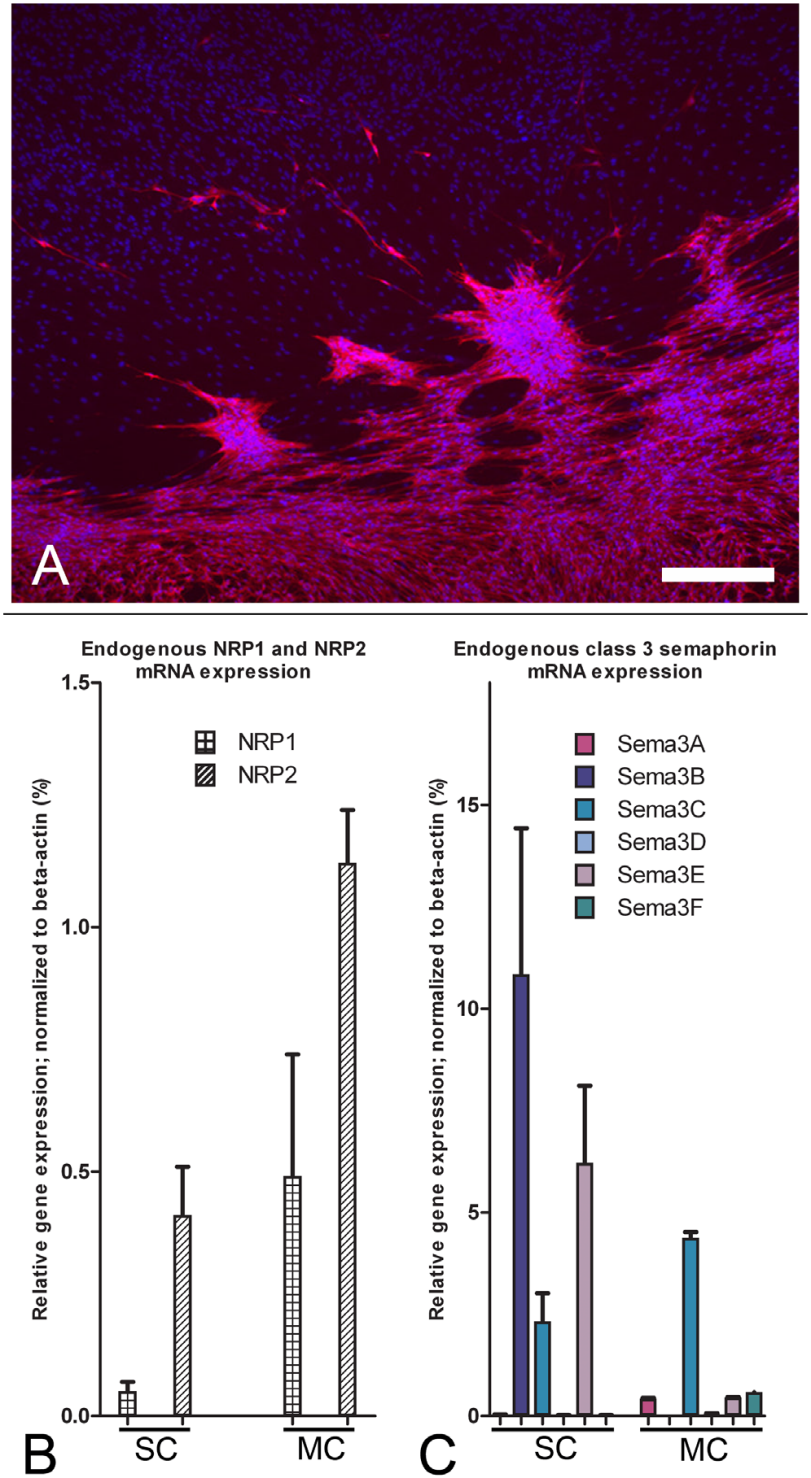
Schwann cells form clusters when confronted with meningeal cells and these cells express neuropilins and class-3 semaphorins. (A) SCs labeled with S100 (red) form a clear border and form cluster-like aggregates when confronted with MCs (blue nuclei without cellular staining). (B) NRP1 and NRP2 are expressed in SCs and in MCs. SCs express 8 fold more NRP2 than NRP1. MCs express ∼2 fold more NRP2 than NRP1. MCs express 10 fold more NRP1 and almost 3 fold more NRP2 than SCs. Scalebar = 250 µm (C) endogenous Sema3A–F expression of SCs and MCs. SCs express predominantly Sema3B, C and E. MC express predominantly Sema3C and at 10 fold lower levels Sema3A, E and F. These results represent the average mRNA levels of 3 independent SC and MC cultures. Error bars indicate the standard error of the mean (SEM).

When SCs encounter MCs in culture they aggregate, align in clusters and show almost no intermingling (Figure 1A) [23]. Class 3 semaphorins can mediate cell repulsion and migration and are possibly involved in this interaction [27], [30]. Both SCs and MCs express the class 3 semaphorin receptors NRP1 and NRP2 (Figure 1B). MCs have a 10 fold higher expression of NRP1 and a 3 fold higher expression of NRP2 than SCs (Figure 1B). NRP2 is more abundant than NRP1 in both SCs and MCs (Figure 1B). SCs and MCs differ in their class 3 semaphorin expression profile: SCs predominantly express Sema3B, C and E while MCs express predominantly Sema3C and to a lesser extend Sema3A, E and F.

### NRP1 and NRP2 mediate Schwann cell/meningeal cell interaction in vitro

**Figure 2:**
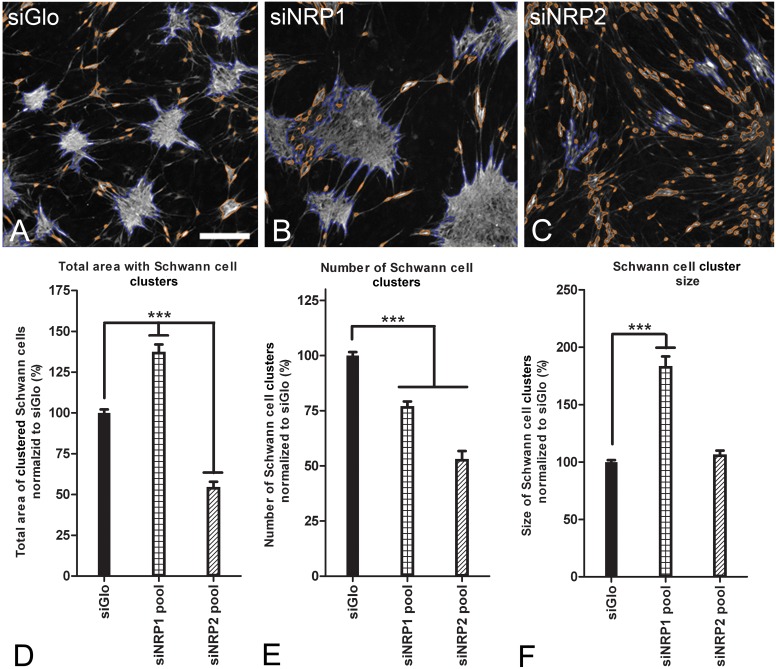
Knockdown of NRP1 increases while knockdown of NRP2 decreases formation of Schwann cell clusters. (A–C) Representative SC clusters when co-cultured with MCs for 5 days after a transfection with siGlo, siNRP1 pool or siNRP2 pool respectively. SCs are shown in grey (S100 labeling). MCs are not labeled. Valid cell clusters were outlined with a blue line. Invalid clusters were outlined with an orange line. Scalebar = 250 µm. (D) The total area of SC clusters per well after knockdown of NRP1 or NRP2. There was a significant increase in total area of SC clusters after knockdown of NRP1 (137%) while in contrast, there was a strong decrease of total area of SC clusters after knockdown of NRP2 (54%). (E) The number of valid clusters per well significantly decreases to 77% after knockdown of NRP1 and 53% after knockdown of NRP2. (F) Cluster size after knockdown of NRP1 increases strongly to 183% (p<0.001) when compared to the siGlo condition, while the average SC cluster size after knockdown of NRP2 does not change. Values are normalized to the siGlo condition and represent the averages of all wells of 6 separate experiments. Error bars indicate the SEM.

SCs aggregate into clusters in SC/MC co-cultures [23]. The significance of NRP1 and NRP2 expression in SCs for this formation of MC-induced SC clusters was examined in loss of function interaction assays. A knockdown of NRP1 resulted in an increase of SC clusters and a clearly visible decrease of loosely aligned SCs between the clusters (Figure 2A and B). In contrast, a knockdown of NRP2 resulted in a strong decrease of SC clusters and a clear increase in SCs that were spread out over the well surface and were loosely aligned in typical columnar arrays (Figure 2A and C). Knockdown of NRP1 or NRP2 changed the total surface area of the wells covered by SC clusters to 137% and 54% of control level respectively (p<0.001; Figure 2D). The increase in cluster surface area after knockdown of NRP1 was the result of a strong increase in cluster size (183%; Figure 2F) while the decrease after knockdown of NRP2 resulted in a strong decline in the number of clusters (53%; Figure 2E). Knockdown of NRP1 also resulted in a relatively small but significant decline in the number of clusters (77% of control levels; Figure 2E).

### Validation of the observed effects of NRP1 and NRP2

**Figure 3:**
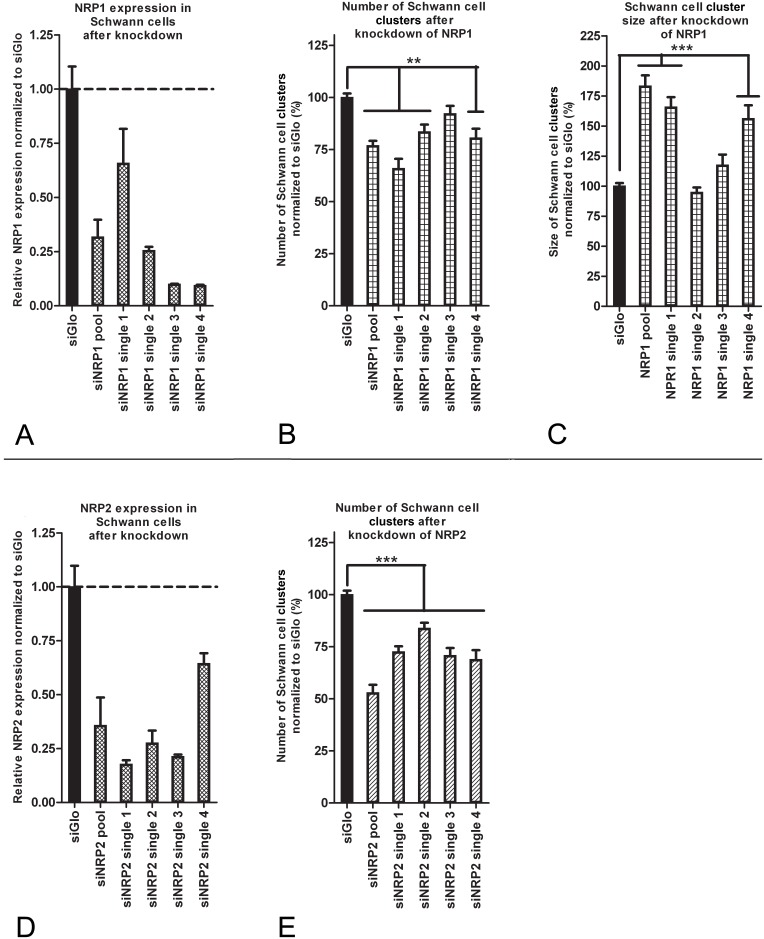
Validation of NRP1 and NRP2 knockdown effects on SC cluster formation. (A) mRNA levels of NRP1 were decreased in SCs after transfection with the siRNA pool or either of the 4 single siRNAs. Expression levels were ranging from 9 to 66% of endogenous expression measured in the siGlo condition. Knockdown was significant for all conditions (p<0.001) except for siNRP1 single 1 (p<0.12). The value presented for the siNRP1 pool is the result of 4 different experiments. The values for the siNRP1 singles are the result of 1 experiment with 4 technical replicates. The siGlo condition presented in the graph is the control for the single siRNA conditions. (B) Three single siRNAs confirm the significant decrease in the number of clusters which is observed with the siNRP1 pool (p<0.01). (C) Two of the single siRNAs confirm the increase of cluster size observed with the siNRP1 pool (p<0.001). All values are normalized to the siGlo condition. The presented values represent the average of 3 separate experiments. The differential effects of siNRP1 single 2 and 3 may be the result of off-target effects (D) mRNA levels of NRP2 were decreased in SCs after transfection with the siRNA pool or either of the 4 single siRNAs. Expression levels were ranging from 18 to 64% of endogenous expression measured in the siGlo condition. Knockdown was significant for all conditions (p<0.05). The value presented for the siNRP2 pool is the result of 4 different experiments. The values for the siNRP1 singles are the result of 1 experiment with 4 technical replicates. The siGlo condition presented in the graph is the control for the single siRNA conditions. (E) All 4 single siRNAs confirm the significant decrease in the number of clusters which is observed with the siNRP2 pool (p<0.01). The presented values represent the average of 3 separate experiments. Error bars indicate the SEM.

The results presented above were obtained with a pool of 4 siRNAs that target either NRP1 or NRP2. The rationale behind the use of pools of 4 siRNAs is that the concentrations of each individual siRNA can be lowered. Consequently, a potential off-target effect of one of the siRNAs will be less severe. The on-target effect will not be decreased since all 4 individual siRNAs target the same gene. Since the 4 single duplexes are distinct sequences, knockdown results of the pools are considered confirmed when the effects can be reproduced by at least 2 of the 4 individual siRNAs [34], [35]. To examine the possibility that the observed effects in the interaction assay were caused by off-target effects, the effects of the 4 single siRNAs from the pools were studied individually. First, the knockdown of NRP1 or NRP2 was determined at the mRNA level and then the effects on cluster formation were examined. SCs that were transfected with the siNRP1 pool or the siNRP1 single siRNAs reached confluent monolayers after 3 days and expression levels of NRP1 varying from 9 to 66% of endogenous expression levels were observed (Figure 3A). The observed decrease in the number of SC clusters was confirmed with 3 of the single siRNA’s (Figure 3B; p<0.01). The observed increase in cluster size was confirmed with 2 of the single siRNA’s (Figure 3C; p<0.001). SCs that were transfected with the siNRP2 pool or the siNRP2 single siRNAs also reached confluent monolayers after 3 days and expression levels of NRP2 varied from 18 to 64% of control levels (Figure 3D). The decrease in the number of SC clusters was confirmed with all 4 single siRNAs (Fig. 3E; p<0.001).

### Knockdown of NRP2 expression causes increased mRNA expression of NRP1

**Figure 4:**
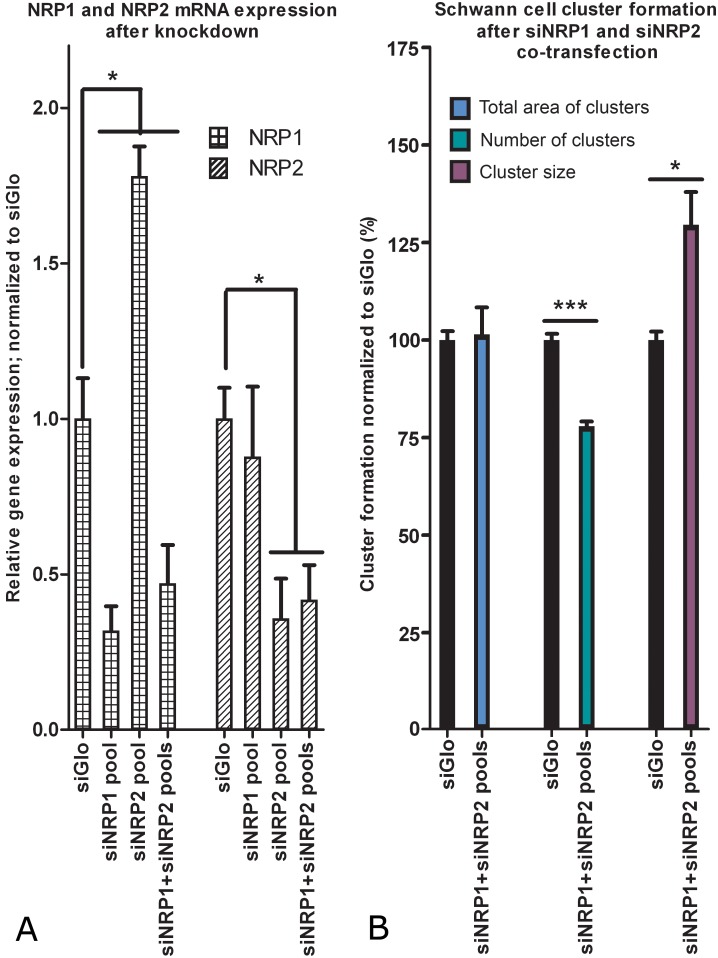
Knockdown of NRP2 expression causes increased mRNA expression of NRP1, while simultaneous knockdown of both NRPs has no effect on the total area of SC clusters. SCs were transfected with the siNRP1 pool, the siNRP2 pool or a combination of both. (A) After 5 days the measured NRP1 mRNA expression levels were 32, 178, and 47% respectively when compared to the siGlo condition. This indicates NRP1 mRNA upregulation after knockdown of NRP2. mRNA expression levels of NRP2 after knockdown with the siNRP1 pool, the siNRP2 pool or a combination of both were 88, 36 and 42% of endogenous levels respectively. The presented values are averages of 4 independent SC transfections (p<0.05). (B) A simultaneous transfection with the siNRP1 and siNRP2 pool has no effect on the total area of SC clusters. There is a small but significant decrease in the number of clusters (78%; p<0.001) and a moderate increase in cluster size (130%; p<0.05). The presented values are averages of 3 independent experiments (P<0.05). Error bars indicate the SEM.

To investigate if the siRNA pools also affected the expression of the NRP that was not targeted by the pool, Relative gene expression was analyzed for both NRP1 and -2 after transfections with the siRNA pools targeting either NRP1, NRP2 or both. Knockdown of NRP2 resulted in an increase in mRNA expression of NRP1 to 178% of control levels (Figure 4A). A comparable increase of NRP1 expression after a knockdown of NRP2 was confirmed by 3 single siRNAs targeting NRP2 (data not shown). NRP2 mRNA expression did not change after knockdown of NRP1. After a co-transfection with the siNRP1 and the siNRP2 pool, NRP1 expression was decreased to 42% and NRP2 expression to 47% of control levels. Combined knock-down of NRP1 and NRP2 resulted in a relatively small increase in SC cluster size and a small decrease in cluster numbers (Figure 4B). No visible difference was observed in the capacity of SCs to loosely align. The combination of these effects resulted in an unchanged total surface area covered by SC clusters.

## Discussion

SCs that are implanted in the lesioned spinal cord form cellular clusters that exhibit only limited migration and intermingling with the reactive astrocytes and MCs that form the scar [10], [14]–[16]. This process can be studied and manipulated in cell culture. For instance in co-cultures of SCs and MCs, SCs form non-overlapping cell clusters with well-defined boundaries [23]. In this article evidence is provided that NRPs are involved in this process.

Both SCs and MCs express NRP1 and NRP2, while SCs express predominantly Sema3B, C and E and MCs express Sema3A, C, E and F. SC mono-cultures are known to align and form columnar arrays. We now show that in SC-MC co-cultures, siRNA mediated knockdown of NRP2 in SCs decreases the formation of SC clusters while the SCs maintain their capacity to align in bands of Büngner like columnar arrays. This indicates that in SC-MC co-cultures, SC aggregation is dependent on NRP2 while SC alignment is governed by another mechanism. This hypothesis is supported by the observation that in rats deficient of NRP2, SC alignment in the bands of Büngner distal to a peripheral nerve lesion is similar to SC alignment in wild type rats [36].

The results described here suggest that a reduction in NRP2 expression may increase the capacity of implanted SCs to integrate in the scar in the lesioned spinal cord while they remain capable of forming bands of Büngner-like structures after implantation that can direct axonal growth through the injured area [36]. MC-conditioned medium has been shown to induce SC aggregation [23]. This indicates that a soluble factor is involved in the formation of SC clusters in the presence of MCs. Knockdown of NRP2 decreases SC sensitivity to the repulsive effects of class 3 semaphorins expressed by MCs and SCs such as Sema3F, which is known to regulate migration and/or adhesion by NRP2 signaling in several different cell types including neural crest cells [37]–[42]. There are strong indications in neurons that both NRP2 and plexinA3 are necessary for Sema3F signal transduction [43]. Since neonatal SCs have been shown to express both NRP2 and plexin A3 [31], [43], it is possible that a similar interaction mechanisms is required to mediate MC induced SC aggregation. The observed increase in NRP1 mRNA expression after NRP2 knockdown suggests an increase in NRP1 protein levels which could amplify this effect because NRP1 can act as a scavenger for Sema3B, C and F [41], [44]. These semaphorins do bind to NRP1 but binding to NRP1 does not transduce repulsive signals [41], [44], thereby potentially causing a further reduction of NRP2 mediated signaling.

Unexpectedly, knockdown of NRP1 expression resulted in a significant increase in SC cluster size. The knockdown of NRP1 can result in a diminished number of class 3 semaphorin binding sites on SCs and as a consequence an enhanced concentration of class 3 semaphorins in the culture medium. These semaphorins can potentially signal through the NRP2 receptor and this could cause the observed increase in MC induced SC aggregation following NRP1 knockdown.

NRP1 expression has been shown to promote cell adhesion [45], [46] and NRPs are capable of homo- and heterotypic interactions in cis and trans [47]. Therefore the knockdown of NRPs in SCs may modify their adherence to and interaction with other SCs and/or MCs.

## References

[1] David S, Aguayo AJ (1981) Axonal elongation into peripheral nervous system “bridges” after central nervous system injury in adult rats. Science 214: 931–933. 6171034 10.1126/science.6171034

[2] Richardson PM, McGuinness UM, Aguayo AJ (1980) Axons from CNS neurons regenerate into PNS grafts. Nature 284: 264–265. 7360259 10.1038/284264a0

[3] Bunge MB (2008) Novel combination strategies to repair the injured mammalian spinal cord. J Spinal Cord Med 31: 262–269. 18795474 10.1080/10790268.2008.11760720 PMC2565567

[4] Morrissey TK, Kleitman N, Bunge RP (1991) Isolation and functional characterization of Schwann cells derived from adult peripheral nerve. J Neurosci 11: 2433–2442. 1869923 10.1523/JNEUROSCI.11-08-02433.1991 PMC6575499

[5] Guest JD, Rao A, Olson L, Bunge MB, Bunge RP (1997) The ability of human Schwann cell grafts to promote regeneration in the transected nude rat spinal cord. Exp Neurol 148: 502–522. 9417829 10.1006/exnr.1997.6693

[6] Saberi H, Moshayedi P, Aghayan HR, Arjmand B, Hosseini SK, et al (2008) Treatment of chronic thoracic spinal cord injury patients with autologous Schwann cell transplantation: an interim report on safety considerations and possible outcomes. Neurosci Lett 443: 46–50. 18662744 10.1016/j.neulet.2008.07.041

[7] Bregman BS, Coumans JV, Dai HN, Kuhn PL, Lynskey J, et al (2002) Transplants and neurotrophic factors increase regeneration and recovery of function after spinal cord injury. Prog Brain Res 137: 257–273. 12440372 10.1016/s0079-6123(02)37020-1

[8] Duncan ID, Aguayo AJ, Bunge RP, Wood PM (1981) Transplantation of rat Schwann cells grown in tissue culture into the mouse spinal cord. J Neurol Sci 49: 241–252. 7217983 10.1016/0022-510x(81)90082-4

[9] Golden KL, Pearse DD, Blits B, Garg MS, Oudega M, et al (2007) Transduced Schwann cells promote axon growth and myelination after spinal cord injury. Exp Neurol 207: 203–217. 17719577 10.1016/j.expneurol.2007.06.023 PMC3513343

[10] Oudega M, Moon LD, de Almeida Leme RJ (2005) Schwann cells for spinal cord repair. Braz J Med Biol Res 38: 825–835. 15933775 10.1590/s0100-879x2005000600003

[11] Oudega M, Xu XM (2006) Schwann cell transplantation for repair of the adult spinal cord. J Neurotrauma 23: 453–467. 16629629 10.1089/neu.2006.23.453

[12] Tuszynski MH, Weidner N, McCormack M, Miller I, Powell H, et al (1998) Grafts of genetically modified Schwann cells to the spinal cord: survival, axon growth, and myelination. Cell Transplant 7: 187–196. 9588600 10.1177/096368979800700213

[13] Garcia-Alias G, Lopez-Vales R, Fores J, Navarro X, Verdu E (2004) Acute transplantation of olfactory ensheathing cells or Schwann cells promotes recovery after spinal cord injury in the rat. J Neurosci Res 75: 632–641. 14991839 10.1002/jnr.20029

[14] Pearse DD, Sanchez AR, Pereira FC, Andrade CM, Puzis R, et al (2007) Transplantation of Schwann cells and/or olfactory ensheathing glia into the contused spinal cord: Survival, migration, axon association, and functional recovery. Glia 55: 976–1000. 17526000 10.1002/glia.20490

[15] Fairless R, Frame MC, Barnett SC (2005) N-cadherin differentially determines Schwann cell and olfactory ensheathing cell adhesion and migration responses upon contact with astrocytes. Mol Cell Neurosci 28: 253–263. 15691707 10.1016/j.mcn.2004.09.009

[16] Iwashita Y, Fawcett JW, Crang AJ, Franklin RJ, Blakemore WF (2000) Schwann cells transplanted into normal and X-irradiated adult white matter do not migrate extensively and show poor long-term survival. Exp Neurol 164: 292–302. 10915568 10.1006/exnr.2000.7440

[17] Luo J, Bo X, Wu D, Yeh J, Richardson PM, et al (2011) Promoting survival, migration, and integration of transplanted Schwann cells by over-expressing polysialic acid. Glia 59: 424–434. 21264949 10.1002/glia.21111

[18] Lakatos A, Barnett SC, Franklin RJ (2003) Olfactory ensheathing cells induce less host astrocyte response and chondroitin sulphate proteoglycan expression than Schwann cells following transplantation into adult CNS white matter. Exp Neurol 184: 237–246. 14637095 10.1016/s0014-4886(03)00270-x

[19] Lakatos A, Franklin RJ, Barnett SC (2000) Olfactory ensheathing cells and Schwann cells differ in their in vitro interactions with astrocytes. Glia 32: 214–225. 11102963 10.1002/1098-1136(200012)32:3<214::aid-glia20>3.0.co;2-7

[20] Afshari FT, Kwok JC, Fawcett JW (2010) Astrocyte-produced ephrins inhibit schwann cell migration via VAV2 signaling. J Neurosci 30: 4246–4255. 20335460 10.1523/JNEUROSCI.3351-09.2010 PMC6634495

[21] Afshari FT, Kwok JC, White L, Fawcett JW (2010) Schwann cell migration is integrin-dependent and inhibited by astrocyte-produced aggrecan. Glia 58: 857–869. 20155822 10.1002/glia.20970

[22] Wilby MJ, Muir EM, Fok-Seang J, Gour BJ, Blaschuk OW, et al (1999) N-Cadherin inhibits Schwann cell migration on astrocytes. Mol Cell Neurosci 14: 66–84. 10433818 10.1006/mcne.1999.0766

[23] Franssen EH, Roet KC, de Bree FM, Verhaagen J (2009) Olfactory ensheathing glia and Schwann cells exhibit a distinct interaction behavior with meningeal cells. J Neurosci Res 87: 1556–1564. 19140223 10.1002/jnr.21979

[24] Chen H, Chedotal A, He Z, Goodman CS, Tessier-Lavigne M (1997) Neuropilin-2, a novel member of the neuropilin family, is a high affinity receptor for the semaphorins Sema E and Sema IV but not Sema III. Neuron 19: 547–559. 9331348 10.1016/s0896-6273(00)80371-2

[25] Kolodkin AL, Levengood DV, Rowe EG, Tai YT, Giger RJ, et al (1997) Neuropilin is a semaphorin III receptor. Cell 90: 753–762. 9288754 10.1016/s0092-8674(00)80535-8

[26] Takahashi T, Fournier A, Nakamura F, Wang LH, Murakami Y, et al (1999) Plexin-neuropilin-1 complexes form functional semaphorin-3A receptors. Cell 99: 59–69. 10520994 10.1016/s0092-8674(00)80062-8

[27] De Winter F, Oudega M, Lankhorst AJ, Hamers FP, Blits B, et al (2002) Injury-induced class 3 semaphorin expression in the rat spinal cord. Exp Neurol 175: 61–75. 12009760 10.1006/exnr.2002.7884

[28] Niclou SP, Franssen EH, Ehlert EM, Taniguchi M, Verhaagen J (2003) Meningeal cell-derived semaphorin 3A inhibits neurite outgrowth. Mol Cell Neurosci 24: 902–912. 14697657 10.1016/s1044-7431(03)00243-4

[29] Pasterkamp RJ, Giger RJ, Ruitenberg MJ, Holtmaat AJ, De Wit J, et al (1999) Expression of the gene encoding the chemorepellent semaphorin III is induced in the fibroblast component of neural scar tissue formed following injuries of adult but not neonatal CNS. Mol Cell Neurosci 13: 143–166. 10192772 10.1006/mcne.1999.0738

[30] Kaneko S, Iwanami A, Nakamura M, Kishino A, Kikuchi K, et al (2006) A selective Sema3A inhibitor enhances regenerative responses and functional recovery of the injured spinal cord. Nat Med 12: 1380–1389. 17099709 10.1038/nm1505

[31] Ara J, Bannerman P, Shaheen F, Pleasure DE (2005) Schwann cell-autonomous role of neuropilin-2. J Neurosci Res 79: 468–475. 15635621 10.1002/jnr.20370

[32] Ara J, Bannerman P, Hahn A, Ramirez S, Pleasure D (2004) Modulation of sciatic nerve expression of class 3 semaphorins by nerve injury. Neurochem Res 29: 1153–1159. 15176472 10.1023/b:nere.0000023602.72354.82

[33] Scarlato M, Ara J, Bannerman P, Scherer S, Pleasure D (2003) Induction of neuropilins-1 and -2 and their ligands, Sema3A, Sema3F, and VEGF, during Wallerian degeneration in the peripheral nervous system. Exp Neurol 183: 489–498. 14552889 10.1016/s0014-4886(03)00046-3

[34] Chan EY, Kir S, Tooze SA (2007) siRNA screening of the kinome identifies ULK1 as a multidomain modulator of autophagy. J Biol Chem 282: 25464–25474. 17595159 10.1074/jbc.M703663200

[35] Echeverri CJ, Beachy PA, Baum B, Boutros M, Buchholz F, et al (2006) Minimizing the risk of reporting false positives in large-scale RNAi screens. Nat Methods 3: 777–779. 16990807 10.1038/nmeth1006-777

[36] Bannerman P, Ara J, Hahn A, Hong L, McCauley E, et al (2008) Peripheral nerve regeneration is delayed in neuropilin 2-deficient mice. J Neurosci Res 86: 3163–3169. 18615644 10.1002/jnr.21766 PMC2574585

[37] Bielenberg DR, Hida Y, Shimizu A, Kaipainen A, Kreuter M, et al (2004) Semaphorin 3F, a chemorepulsant for endothelial cells, induces a poorly vascularized, encapsulated, nonmetastatic tumor phenotype. J Clin Invest 114: 1260–1271. 15520858 10.1172/JCI21378 PMC524226

[38] Favier B, Alam A, Barron P, Bonnin J, Laboudie P, et al (2006) Neuropilin-2 interacts with VEGFR-2 and VEGFR-3 and promotes human endothelial cell survival and migration. Blood 108: 1243–1250. 16621967 10.1182/blood-2005-11-4447

[39] Gammill LS, Gonzalez C, Bronner-Fraser M (2007) Neuropilin 2/semaphorin 3F signaling is essential for cranial neural crest migration and trigeminal ganglion condensation. Dev Neurobiol 67: 47–56. 17443771 10.1002/dneu.20326

[40] Gammill LS, Gonzalez C, Gu C, Bronner-Fraser M (2006) Guidance of trunk neural crest migration requires neuropilin 2/semaphorin 3F signaling. Development 133: 99–106. 16319111 10.1242/dev.02187

[41] Nasarre P, Kusy S, Constantin B, Castellani V, Drabkin HA, et al (2005) Semaphorin SEMA3F has a repulsing activity on breast cancer cells and inhibits E-cadherin-mediated cell adhesion. Neoplasia 7: 180–189. 15802023 10.1593/neo.04481 PMC1501131

[42] Schwarz Q, Maden CH, Davidson K, Ruhrberg C (2009) Neuropilin-mediated neural crest cell guidance is essential to organise sensory neurons into segmented dorsal root ganglia. Development 136: 1785–1789. 19386662 10.1242/dev.034322 PMC2680105

[43] Cheng HJ, Bagri A, Yaron A, Stein E, Pleasure SJ, et al (2001) Plexin-A3 mediates semaphorin signaling and regulates the development of hippocampal axonal projections. Neuron 32(2): 249–63. 11683995 10.1016/s0896-6273(01)00478-0

[44] Takahashi T, Nakamura F, Jin Z, Kalb RG, Strittmatter SM (1998) Semaphorins A and E act as antagonists of neuropilin-1 and agonists of neuropilin-2 receptors. Nat Neurosci 1: 487–493. 10196546 10.1038/2203

[45] Shimizu M, Murakami Y, Suto F, Fujisawa H (2000) Determination of cell adhesion sites of neuropilin-1. J Cell Biol 148: 1283–1293. 10725340 10.1083/jcb.148.6.1283 PMC2174302

[46] Takagi S, Kasuya Y, Shimizu M, Matsuura T, Tsuboi M, et al (1995) Expression of a cell adhesion molecule, neuropilin, in the developing chick nervous system. Dev Biol 170: 207–222. 7601310 10.1006/dbio.1995.1208

[47] Chen H, He Z, Bagri A, Tessier-Lavigne M (1998) Semaphorin-neuropilin interactions underlying sympathetic axon responses to class III semaphorins. Neuron 21: 1283–1290. 9883722 10.1016/s0896-6273(00)80648-0

